# Supplementary material for: The global distribution patterns of alien vertebrate richness in mountains
Source: Nat Commun. 2025 Feb 26;16:1977. doi: 10.1038/s41467-025-57214-w (PMC11862207; doi:10.1038/s41467-025-57214-w)
Supplement: Supplementary file 1 — Supplementary Information [file 41467_2025_57214_MOESM1_ESM.pdf]

Supplementary information for:

The global distribution patterns of alien vertebrate richness in mountains

Adrián García-Rodríguez*[1], Bernd Lenzner[1], Julián A. Velasco[2], Anna Schertler[1], Ali Omer[1], Hanno Seebens[3,4], César Capinha[5,6], Belinda Gallardo[7], Stefan Dullinger[8], & Franz Essl[1]

[1] Division of BioInvasions, Global Change & Macroecology, Department of Botany and Biodiversity Research, University of Vienna, Rennweg 14, AT 1030, Vienna, Austria
[2] Instituto de Ciencias de la Atmósfera y Cambio Climático, Universidad Nacional Autónoma de México, Ciudad Universitaria, Ciudad de México, México
[3] Senckenberg Biodiversity and Climate Research Centre, Frankfurt, Germany
[4] Department of Animal Ecology & Systematics, Justus Liebig University Giessen, Giessen, Germany
[5] Centre of Geographical Studies, Institute of Geography and Spatial Planning, University of Lisbon, Lisbon, Portugal
[6] Associate Laboratory TERRA, Lisbon, Portugal
[7] Instituto Pirenaico de Ecología, CSIC. Avda. Montañana 1005, 50192 Zaragoza, Spain
[8] Division of Biodiversity Dynamics and Conservation, Department of Botany and Biodiversity Research, University of Vienna, Rennweg 14, AT 1030, Vienna, Austria

3 Tables

5 Figures

Supplementary Table 1. Definitions of the protected area categories of management analyzed according to the International Union for the Conservation of Nature (IUCN)

Category	Name	Definition
Ia	Strict Nature Reserve	Strictly protected for biodiversity and also possibly geological/ geomorphological features, where human visitation, use and impacts are controlled and limited to ensure protection of the conservation values
Ib	Wilderness area	Usually large unmodified or slightly modified areas, retaining their natural character and influence, without permanent or significant human habitation, protected and managed to preserve their natural condition
II	National Park	Large natural or near-natural areas protecting large-scale ecological processes with characteristic species and ecosystems, which also have environmentally and culturally compatible spiritual, scientific, educational, recreational and visitor opportunities
III	Natural Monument or feature	Areas set aside to protect a specific natural monument, which can be a landform, sea mount, marine cavern, geological feature such as a cave, or a living feature such as an ancient grove
IV	Habitat or Species Management Area	Areas to protect particular species or habitats, where management reflects this priority. Many will need regular, active interventions to meet the needs of particular species or habitats, but this is not a requirement of the category
V	Protected Landscape	Where the interaction of people and nature over time has produced a distinct character with significant ecological, biological, cultural, and scenic value: and where safeguarding the integrity of this interaction is vital to protecting and sustaining the area and its associated nature conservation and other values
VI	Protected Area with sustainable use of natural resources	Areas that conserve ecosystems, together with associated cultural values and traditional natural resource management systems. Generally large, mainly in a natural condition, with a proportion under sustainable natural resource management and where low-level non-industrial natural resource use compatible with nature conservation is seen as one of the main aims

Supplementary Table 2. Lists of the top ten mountains with the highest numbers of alien vertebrate species both overall and for each group.

Overall			Fishes		
**Mountain**	**Country**	**Richness**	**Mountain**	**Country**	**Richness**
**Cairngorms**	United Kingdom	81	**Mogollon Rim**	United States	15
**The Sperrins**	United Kingdom	79	**Cowee Mountains**	United States	10
**Moorfoot Hills**	United Kingdom	78	**Diablo Range**	United States	9
**Lammermuir Hills**	United Kingdom	77	**Ko'olau Range**	United States	9
**Monadhliath Mountains**	United Kingdom	77	**Mazatzal Mountains**	United States	9
**Cheviot Hills**	United Kingdom	76	**San Rafael Mountains**	United States	9
**San Gabriel Mountains**	United States	48	**East River Mountain**	United States	7
**Santa Monica Mountains**	United States	46	**Great Balsam Mountains**	United States	7
**Snowdonia**	United Kingdom	45	**Laguna Mountains**	Mexico, United States	7
**Black Forest**	Switzerland, Germany	43	**San Bernardino Mountains**	United States	7
**Reptiles**			**Amphibians**		
**Mountain**	**Country**	**Richness**	**Mountain**	**Country**	**Richness**
**Ko'olau Range**	United States	11	**Ko'olau Range**	United States	6
**Puu Hualalai**	United States	9	**Laguna Mountains**	Mexico, United States	5
**Kohala Mountains**	United States	8	**Waianae Range**	United States	5
**Piton des Neiges**	Reunion	8	**Chugoku Mountains**	Japan	4
**Waianae Range**	United States	8	**Digne Prealps**	France	4
**Piton de la Fournaise**	Reunion	7	**Iglit-Baco Mountains**	Philippines	4
**Puu Kukui**	United States	7	**Kohala Mountains**	United States	4
**San Gabriel Mountains**	United States	7	**Long Range Mountains**	Canada	4
**Kamakou**	United States	6	**Massif des Alberes**	Spain, France	4
**Santa Monica Mountains**	United States	6	**San Bernardino Mountains**	United States	4
**Birds**			**Mammals**		
**Mountain**	**Country**	**Richness**	**Mountain**	**Country**	**Richness**
**The Sperrins**	United Kingdom	73	**Main Range**	Australia	19
**Cairngorms**	United Kingdom	72	**Wellington Range**	Australia	14
**Cheviot Hills**	United Kingdom	72	**Illawarra Range**	Australia	13
**Lammermuir Hills**	United Kingdom	71	**Tararua Range**	New Zealand	13
**Moorfoot Hills**	United Kingdom	71	**Cambewarra Range**	Australia	12
**Monadhliath Mountains**	United Kingdom	70	**Jura Mountains**	Switzerland, Germany, France	11
**Black Forest**	Switzerland, Germany	38	**Rimutaka Range**	New Zealand	11
**Hunsrück**	Germany	38	**Snowdonia**	United Kingdom	11
**Kocheler Berge**	Germany	37	**Suggan Buggan Range**	Australia	11
**Sauerland**	Germany	37	**Byadbo Range**	Australia	10

Supplementary Table 3. Flows of vertebrate species between their native realm and the realm where they occur as aliens in mountain ranges. For each realm, we provide the number of total species and the number of species belonging to each vertebrate class. For each taxonomic group, the numbers corresponding to the top three donors and the top three recipients are highlighted in bold.

	All Taxa		Fishes		Amphibians		Birds		Reptiles		Mammals	
**Realm**	**From**	**To**	**From**	**To**	**From**	**To**	**From**	**To**	**From**	**To**	**From**	**To**
Afrotropic	***222***	101	***23***	14	5	3	***130***	38	***22***	16	***42***	30
Australasia	104	***139***	0	6	3	4	60	***65***	18	11	23	***53***
Indo-Malay	***241***	122	*20*	15	*9*	6	***115***	61	***23***	5	***74***	35
Madagascar	18	16	8	4	0	0	0	4	4	0	6	8
Nearctic	***244***	***232***	***75***	***65***	***29***	***23***	***83***	***70***	10	***23***	41	***51***
Neotropics	115	132	18	*18*	*9*	***7***	52	55	19	***21***	17	18
Oceania	2	74	0	12	0	***7***	2	30	0	14	0	11
Palearctic	153	***283***	16	***26***	***19***	***24***	15	***134***	***20***	***26***	***76***	***73***

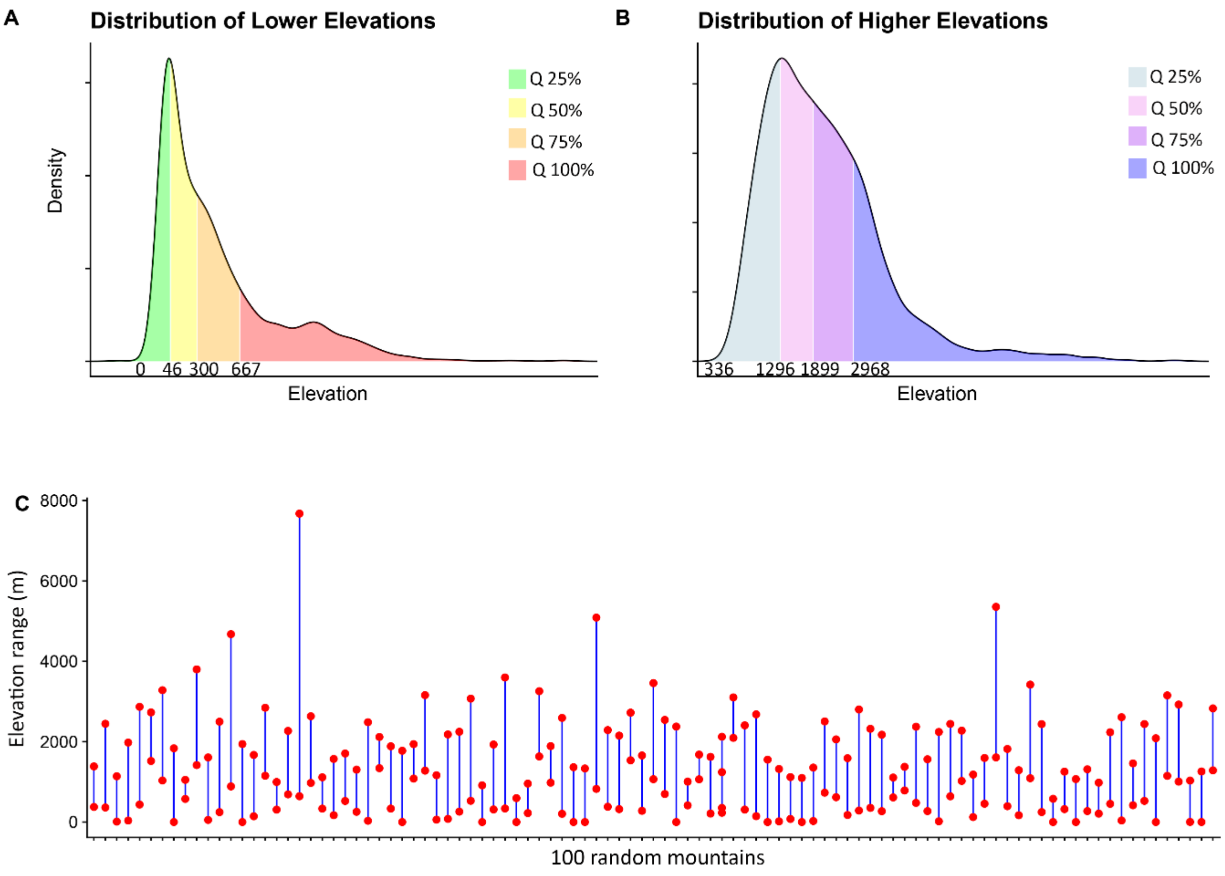

Supplementary Figure 1. Distribution of lower (A) and upper (B) elevation limits covered by the studied mountains. C. shows a sample of the variability of the altitudinal ranges covered by the studied mountains, both in upper and lower limits and in the gradient they span.

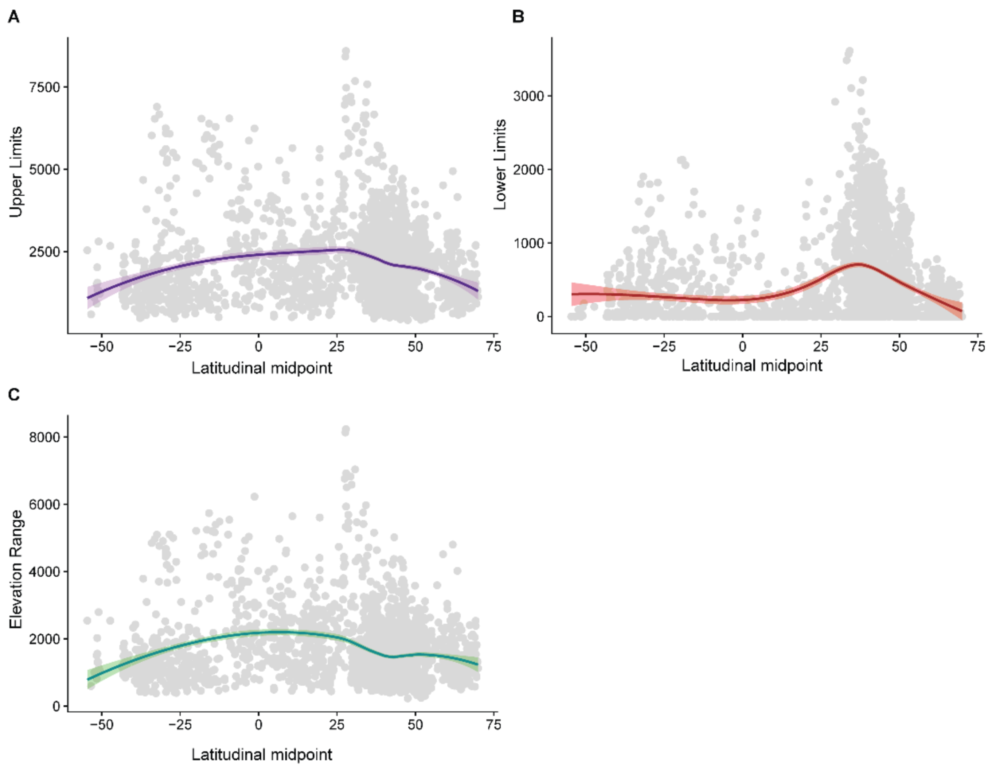

Supplementary Figure 2. Non-linear regressions among the latitudinal midpoints of the studied mountains and their elevation limits (upper in purple, and lower in red), elevation ranges (green) and recorded overall alien richness (orange).

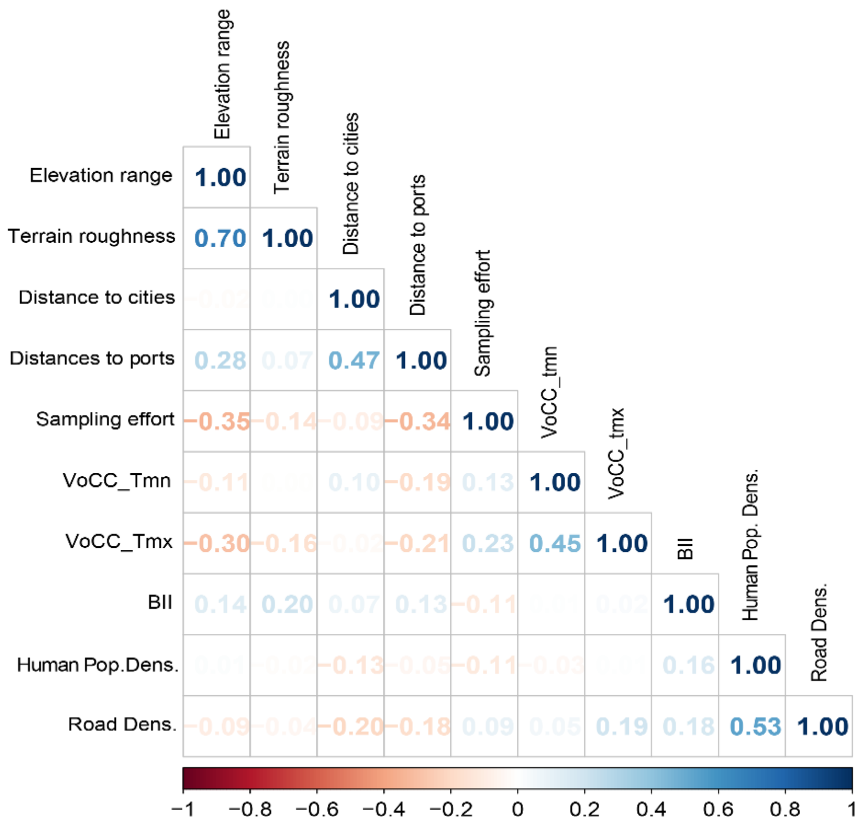

Supplementary Figure 3. Pairwise Pearson correlations among predictor variables.

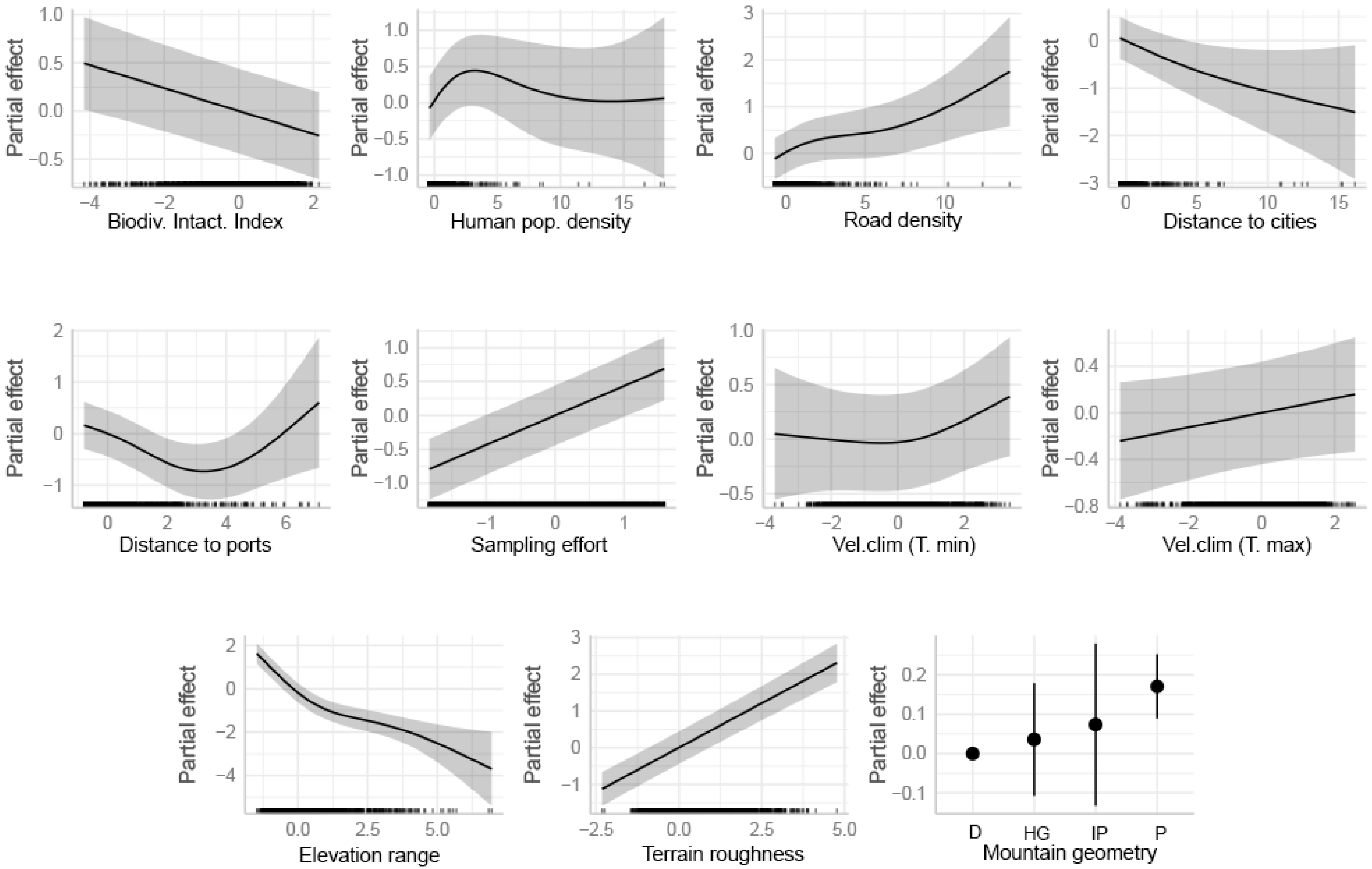

Supplementary Figure 4. Results of a Generalized Additive Mixed Model (GAMM) fitted using the same structure of the GLMM provided in the main text. With both approaches, we obtained similar results in terms of the significance and overall direction of the relationships.

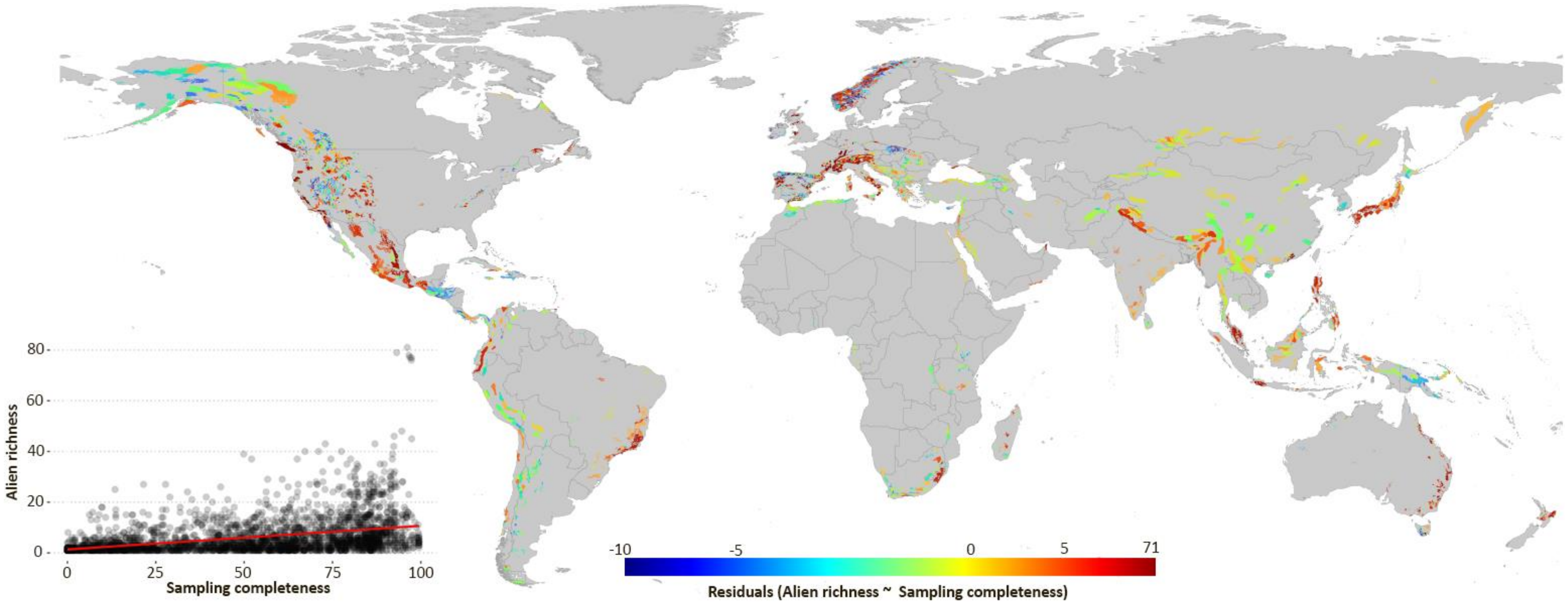

Supplementary Figure 5. Sampling bias-corrected distribution pattern of alien vertebrate richness across the world's mountains. The colored polygons represent the subset of mountains extracted from [1,2] for which our dataset contains records of at least one alien vertebrate species. The map shows the residuals of the regression between alien vertebrate richness and mean sampling completeness estimated for each mountain, as shown in the dispersion plot on the bottom left.

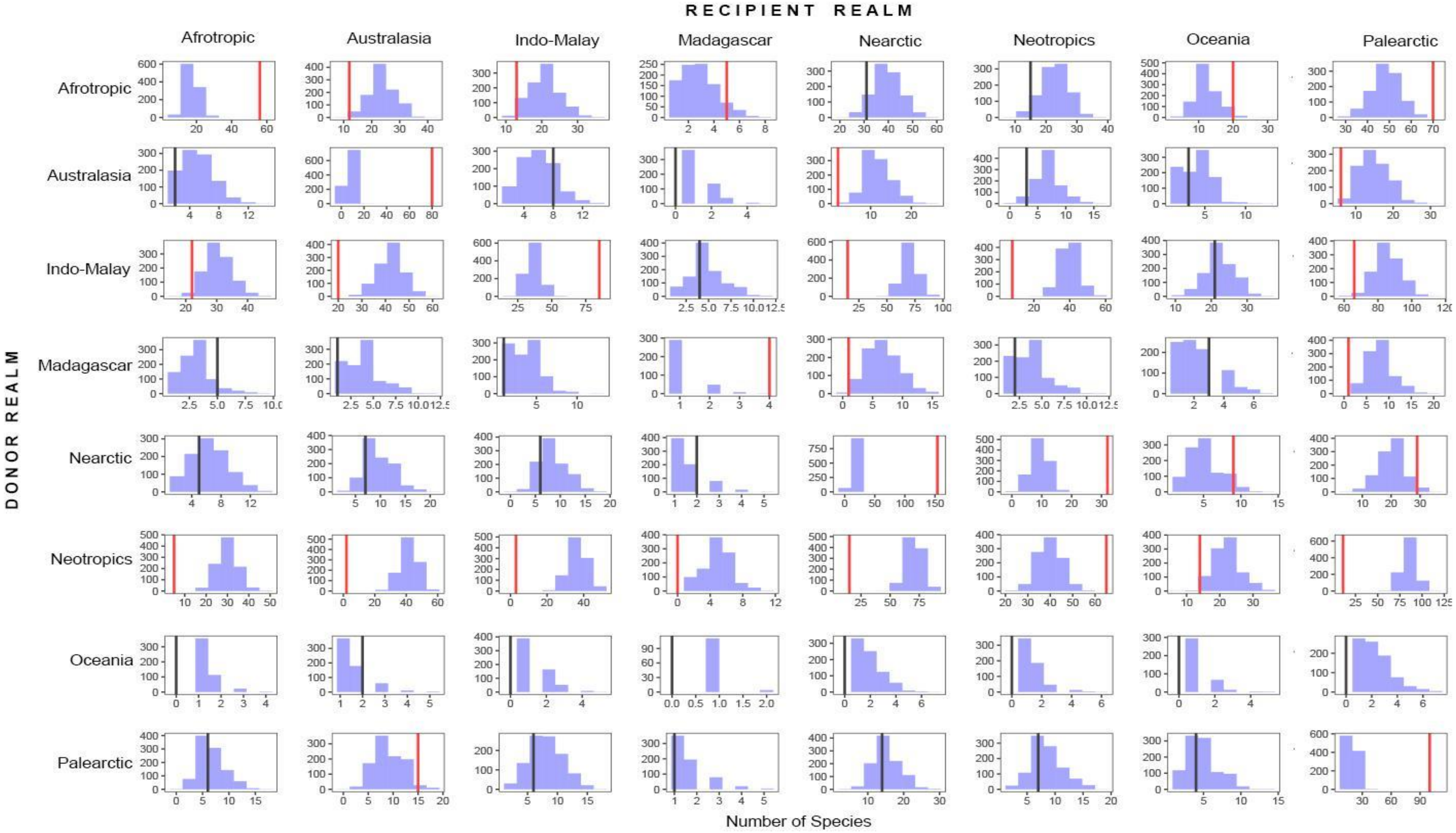

Supplementary Figure 6. Observed and expected number of alien vertebrates exchanged among realms. Histograms show the distribution of 999 random draws performed over datasets containing the full vertebrate pools of each region and using as sampling number the alien vertebrate richness documented in each realm. Red lines show cases where the number of observed species is higher or lower than expected as defined by values higher or lower than the upper and lower 2.5% of the distribution of random draws, respectively.

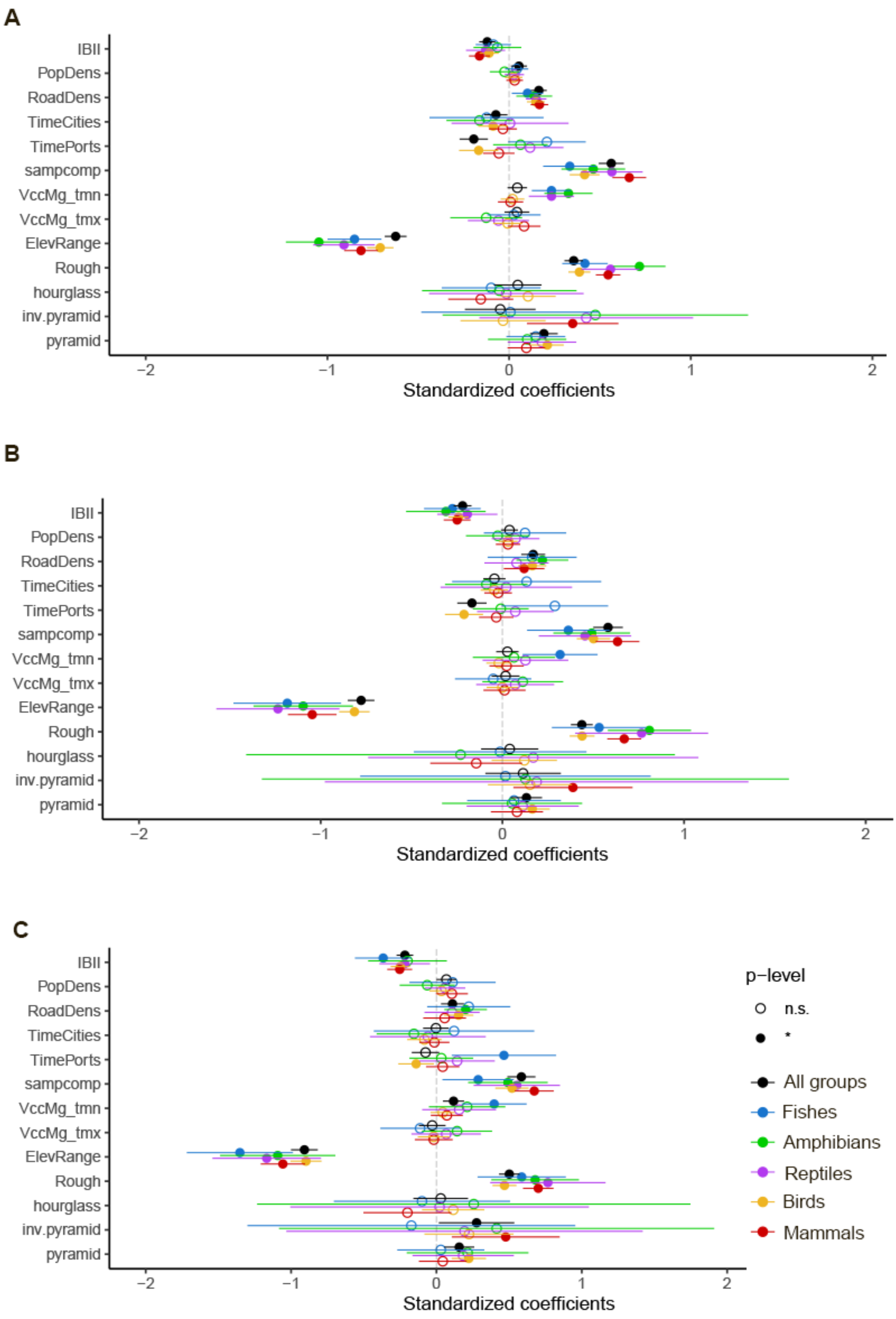

Supplementary Figure 7. Sensitivity analyses for the drivers of alien vertebrate richness in mountains. Shown are the effect sizes for the variables tested as predictors using different subsets of mountains: A. Excluding outliers -i.e. mountains with more than 60 alien vertebrate species documented- (n= 2690 mountains in the overall model). B. Excluding the upper quartile of the distribution of alien vertebrate richness in mountains (n= 1979 mountains in

the overall model). C. Excluding both the upper and lower quartiles of the distribution of alien vertebrate richness in mountains (n= 1005 mountains in the overall model). For each predictor six effect sizes are shown, representing the cross-taxon model (black symbols) and the five taxon-specific models, each in a different color. Values to the left of the zero line depict negative relationships between the response variable and the respective predictor (i.e., alien richness increases as the predictor decreases), and those to the right show positive relationships. Unfilled circles represent variables with non-significant effects. Filled circles effects with statistical significance ($p < 0.05$). Significances for the geometry types are against the baseline level of a diamond-shaped mountain.

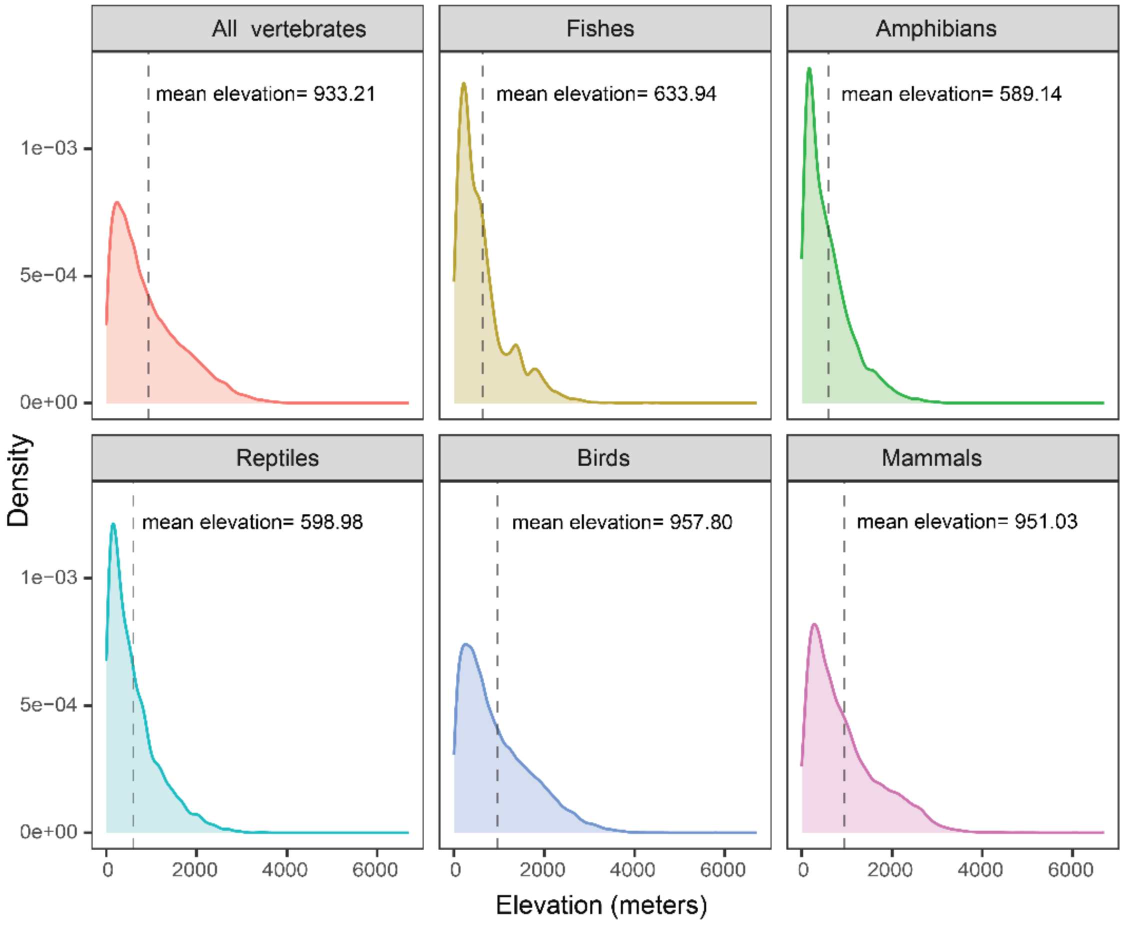

Supplementary Figure 8. Distribution of elevation values of the > 165k occurrences analyzed in this study. Density plots are provided both for the overall dataset and at the taxon level. Dashed lines show the mean value of the extracted elevations for the whole dataset and for each group. The information plotted was extracted from an SRTM elevation layer at 30 arcsec resolution (c. 1x1 km at the equator) using the records here studied. Since these records were obtained from GBIF we expect uncertainties in the elevations, mainly in rough topographies where elevation changes can be strong even in short distances.

REFERENCES

1. Snethlage, M. A. *et al.* A hierarchical inventory of the world's mountains for global comparative mountain science. *Sci Data* 9, 1–14 (2022).

2. Snethlage, M. A. *et al.* GMBA Mountain Inventory v2. *GMBA-EarthEnv* (2022) doi:https://doi.org/10.48601/earthenv-t9k2-1407.
